# RcLS2F – A Novel Fungal Class 1 KDAC Co-repressor Complex in *Aspergillus nidulans*

**DOI:** 10.3389/fmicb.2020.00043

**Published:** 2020-02-04

**Authors:** Ingo Bauer, Silke Gross, Petra Merschak, Leopold Kremser, Betim Karahoda, Özlem Sarikaya Bayram, Beate Abt, Ulrike Binder, Fabio Gsaller, Herbert Lindner, Özgür Bayram, Gerald Brosch, Stefan Graessle

**Affiliations:** ^1^Institute of Molecular Biology, Biocenter, Medical University of Innsbruck, Innsbruck, Austria; ^2^Institute of Clinical Biochemistry, Biocenter, Medical University of Innsbruck, Innsbruck, Austria; ^3^Biology Department, Maynooth University, Maynooth, Ireland; ^4^Institute of Hygiene and Medical Microbiology, Medical University of Innsbruck, Innsbruck, Austria

**Keywords:** RpdA, lysine deacetylase (KDAC), histone deacetylase (HDAC), *Aspergillus nidulans*, Ascomycota, chromatin, co-repressor complex, calcium

## Abstract

The fungal class 1 lysine deacetylase (KDAC) RpdA is a promising target for prevention and treatment of invasive fungal infection. RpdA is essential for survival of the most common air-borne mold pathogen *Aspergillus fumigatus* and the model organism *Aspergillus nidulans*. In *A. nidulans*, RpdA depletion induced production of previously unknown small bioactive substances. As known from yeasts and mammals, class 1 KDACs act as components of multimeric protein complexes, which previously was indicated also for *A. nidulans*. Composition of these complexes, however, remained obscure. In this study, we used tandem affinity purification to characterize different RpdA complexes and their composition in *A. nidulans*. In addition to known class 1 KDAC interactors, we identified a novel RpdA complex, which was termed RcLS2F. It contains ScrC, previously described as suppressor of the transcription factor CrzA, as well as the uncharacterized protein FscA. We show that recruitment of FscA depends on ScrC and we provide clear evidence that Δ*crzA* suppression by ScrC depletion is due to a lack of transcriptional repression caused by loss of the novel RcLS2F complex. Moreover, RcLS2F is essential for sexual development and engaged in an autoregulatory feed-back loop.

## Introduction

Fungi colonize all kinds of habitats, either as free-living forms or associated with other organisms, including commercial crops or animals in symbiotic, commensal, or pathogenic frameworks (Dadachova and Casadevall, 2008). Fungi are also human pathogens that can provoke disease through three major mechanisms: (i) by causing allergic reactions (Barnes, 2019), (ii) by production of mycotoxins (Gruber-Dorninger et al., 2017), and (iii) via superficial, invasive, or systemic infections (e.g., van de Veerdonk et al., 2017; Van Dyke et al., 2019). Further, fungi are important as producers of small bioactive molecules that are exploited biotechnologically through their broad range of antibiotic, antiviral, antitumor, antihypercholesterolemic, and immunosuppressant activities (e.g., penicillins, cephalosporins, statins, or cyclosporine A; Keller, 2019).

As in all eukaryotes, fungal DNA is packaged with histones and non-histone proteins into a complex structure known as chromatin (Brosch et al., 2008). Chromatin structure is tightly regulated to adjust DNA accessibility for proteins involved in processes such as replication, transcription, recombination, and DNA repair (Margueron and Reinberg, 2010). Among the mechanisms impacting chromatin structure, post-translational modification of core histones (and other chromatin proteins) plays an outstanding role in providing specific regulatory signals to trigger the remodeling machinery (Andrews et al., 2016). A major modification of histone tails is the reversible acetylation of distinct lysine residues, catalyzed by histone acetyltransferases and their counterparts, histone deacetylases (Verdin and Ott, 2015). However, proteins other than histones are also subject to reversible acetylation involving the very same deacetylases (Narita et al., 2019), therefore we will use the term lysine deacetylase (KDAC) in this article. Based on sequence similarities, classical KDACs are divided into at least three classes including more than 10 paralogs in mammals (Seto and Yoshida, 2014). In filamentous fungi the situation is less complex. *Aspergillus nidulans*, for instance, has only two class 1 enzymes, RpdA and HosA (Graessle et al., 2000, 2001), and two class 2 KDACs, HdaA, and HosB (Trojer et al., 2003).

While *Aspergillus* strains with deletions of *hdaA*, *hosA*, and *hosB* homologous genes show several (phenotypic) characteristics but are viable (Tribus et al., 2005; Shwab et al., 2007; Lee et al., 2009; Kawauchi and Iwashita, 2014; Pidroni et al., 2018; Lan et al., 2019; Li X. et al., 2019), efforts to generate *rpdA* deletion mutants failed so far. Indeed, we proved that RpdA is essential for growth and development of *A. nidulans* and the human pathogen *Aspergillus fumigatus* (Tribus et al., 2010; Bauer et al., 2016). Very recently, we showed that RpdA is required for virulence of *A. fumigatus* in a murine model for pulmonary aspergillosis (Bauer et al., 2019a). Furthermore, expression-studies with several mutated RpdA fragments revealed that a conserved and fungal-specific C-terminal motif of approximately 12 amino acid residues is required for the biological function of this enzyme (Bauer et al., 2016). Consistent with that, RpdA-depleted *A. nidulans* strains cannot be complemented by yeast and human class 1 KDACs lacking this motif (Bauer et al., 2016). Due to the availability of KDAC inhibitors, some of which even have been approved by the FDA (West and Johnstone, 2014), RpdA can be regarded as druggable antifungal target. Given the high conservation of the catalytic domains of fungal and human class 1 KDACs, however, development of fungal-specific inhibitors is desirable to minimize or even prevent side-effects accompanying their use as antifungals. Since most KDACs are guided to their site of action by associated proteins, blocking of specific protein–protein interactions might be an alternative to the direct inhibition of catalytic activity (Millard et al., 2017). In order to exploit this strategy, it is of utmost importance to learn more about diversity and composition of complexes formed around the catalytically active RpdA.

One previously characterized group of class 1 KDAC complexes conserved from yeasts to mammals are the Sin3 complexes (Adams et al., 2018). In *Saccharomyces cerevisiae*, two major Rpd3/Sin3 containing complexes have been described: Rpd3L and Rpd3S. These complexes contain a conserved core composed of Rpd3, Sin3, and Ume1. Rpd3L mediates gene repression at promoter regions, where it is recruited by sequence specific transcription factors (Carrozza et al., 2005a; Sharma et al., 2007; Takahata et al., 2009; Bosio et al., 2017) and is crucial for transcriptional repression memory (Lee et al., 2018). In addition to promoter regions, Rpd3S locates to open reading frames in the wake of elongating RNA polymerase II and suppresses transcription from cryptic promoters (Carrozza et al., 2005b; Joshi and Struhl, 2005; Keogh et al., 2005). Equivalents of Rpd3L and S complexes, Clr6I and II, have also been identified in fission yeast and similarly are implicated in the regulation of chromatin structure and transcription (Nicolas et al., 2007; Choi et al., 2012; Zilio et al., 2014). A third Rpd3 complex lacking Sin3 and Ume1 is involved in environmental stress response pathways, in particular to oxidative stress (Baker et al., 2013). No clear homolog of the latter complex was found in fission yeast so far.

Here we present the identification and characterization of a novel fungal class 1 KDAC/Sin3 complex, RpdA-SinC-PrwA-LafA-SdsC-ScrC-FscA, termed as RcLS2F. We demonstrate the presence of two poorly characterized proteins in this complex, one of which, ScrC, has previously been linked to the hyphal calcium response and recruits the other novel member, FscA. We show that (i) RcLS2F acts as a transcriptional co-repressor and that (ii) the previously published genetic bypass of CrzA by *scrC* loss of function mutants (Almeida et al., 2013) is linked to RcLS2F deficiency. Furthermore, we provide evidence that (iii) RcLS2F plays an important role as transcriptional co-repressor of *crzA*-regulated genes under conditions devoid of calcium-signaling and that (iv) RcLS2F displays autoregulation.

## Results

### Identification of *A. nidulans* RpdA Complexes by Tandem Affinity Chromatography

**FIGURE 1:**
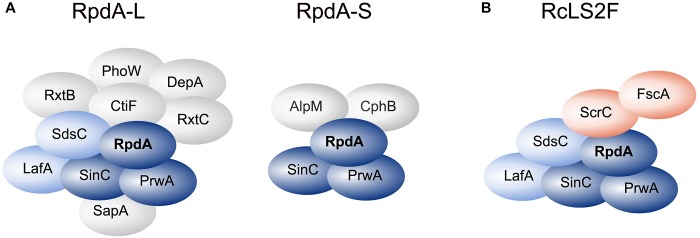
RpdA/SinA complexes in *Aspergillus nidulans*. Schematic representations of complex compositions of RpdA-L and RpdA-S complexes as identified by tandem affinity purification (TAP) coupled to LC-MS/MS protein identification **(A)** and the novel RcLS2F complex **(B)**. The catalytic component RpdA is marked in bold. The RpdA/SinC/PrwA core complex is colored in dark blue. Complex partners shared with RpdA-L and RcLS2F are colored light blue. The defining RcLS2F components, ScrC and FscA, are shown in teal.

Using a strain expressing C-terminally TAP-tagged RpdA (RpdA^TAP^) we conducted tandem affinity purification (TAP; Rigaut et al., 1999) coupled to liquid chromatography-tandem mass spectrometry (LC-MS/MS). Hits that were identified by at least two peptides were searched for homologs in budding and fission yeast databases via BLASTp. This suggested the presence of RpdA complexes corresponding to those described in yeast, i.e., RpdA-L and RpdA-S (Figure 1A and Supplementary Table 1; Lechner et al., 2000; Carrozza et al., 2005a; Nicolas et al., 2007; Baker et al., 2013; Zilio et al., 2014). However, not all known yeast complex members were detected, mainly due to the fact that the *A. nidulans* genome lacks the respective homologs. Based on the presented data, composition of a third RpdA complex homologous to Rpd3μ (Rpd3-Snt2-Ecm5; Baker et al., 2013; McDaniel and Strahl, 2013) is not entirely elucidated yet and requires further investigation (Supplementary Table 1).

Among the remaining hits found in two analyses, two highly ranked proteins, ScrC and AN4022, attracted our attention. To investigate their putative interaction with RpdA, strains expressing C-terminally TAP-tagged versions of these candidates, ScrC^TAP^ and FscA^TAP^, were generated, which were then used as baits for *vice versa* purifications. Results actually confirmed the interaction of both proteins with each other and with RpdA (Table 1). Moreover, SinC, PrwA, and SdsC were highly enriched and also LafA was present in each purification using ScrC^TAP^ and FscA^TAP^ as baits. To exclude co-purification of the detected proteins by interaction with the TAP-tag only, GFP-trap experiments using a strain expressing C-terminally Venus-tagged RpdA, RpdA^Venus^, were performed. Results confirmed those of the TAP experiments (data not shown). Data suggest that ScrC and FscA, together with the RpdA core (RpdA, SinA, PrwA) and two other proteins also found in RpdA-L complexes (LafA and SdsC) constitute a novel RpdA complex in *A. nidulans*. While ScrC was identified in a suppressor screen for Δ*crzA* phenotypes (Almeida et al., 2013), the previously uncharacterized second novel interaction partner (AN4022) was designated FscA (friend of ScrC, see below). Consequently, we have termed the novel complex RpdA core LafA SdsC ScrC FscA (RcLS2F) complex (Figure 1B).

**TABLE 1:** Identification of the RcLS2F complex.

			MW	RpdA	FscA	ScrC
	*A. nidulans*	Accession	[kD]	TAP	TAP	TAP
**Rc**	**RpdA**	**AN4493**	**75.4**	**49.3 (31)**	**55.0 (33)**	**50.5 (31)**
	**SinC**	**AN1546**	**179.5**	**38.2 (46)**	**61.0 (95)**	**57.5 (92)**
	**PrwA**	**AN8187**	**46.2**	**30.6 (9)**	**60.0 (20)**	**46.5 (18)**
**L**	**LafA**	**AN5099**	**44.1**	**10.7 (3)**	**10.8 (4)**	**25.5 (9)**
**S2**	**SdsC**	**AN3178**	**55.6**	**38.9 (13)**	**56.9 (26)**	**58.5 (26)**
	**ScrC**	**AN8823**	**92.7**	**20.1 (12)**	**61.5 (46)**	**62.5 (46)**
**F**	**FscA**	**AN4022**	**83.3**	**30.3 (15)**	**56.0 (46)**	**53.7 (44)**
	RxtB	AN1375	58.0	24.7 (11)		
	RxtC	AN6280	100.1	19.9 (15)	6.0 (4)	8.5 (6)
	SapA	AN6196	24.4	59.1 (11)		
	PhoW	AN5570	67.8	35.7 (15)		
	DepA	AN1453	75.1	21.9 (12)	3.4 (2)	
	CtiF	AN4694	67.4	10.2 (5)	3.0 (2)	
	AlpM	AN1976	38.1	40.4 (11)		5.0 (2)
	CphB	AN7300	92.4	10.5 (6)		
	KdmB	AN8211	193.1	18.8 (20)	3.5 (4)	1.0 (2)
	SntB	AN9517	56.6	23.9 (34)		

*Proteins in ScrC, FscA, and RpdA TAP fractions were identified by LC-MS/MS. Molecular weight (MW) of *A. nidulans* proteins is indicated in kilodalton (kD). The “TAP” columns show mean values of sequence coverage (%) and, in parentheses, of number of identified peptides of two individual purifications for each protein. Empty fields indicate detection below the limit. Refer to Supplementary Data Sheet 1 for complete protein identification data. Constituents of RcLS2F are marked in bold.*

In order to estimate the native size of the RcLS2F complex, fungal extracts of a strain expressing FscA with a C-terminal Venus fluorescent protein tag, FscA^Venus^, were loaded onto a Superose 6 size exclusion column. Western blots of elution fractions probed with antibodies against Venus revealed a FscA peak with retention corresponding to an apparent molecular mass of greater than 1 × 10^6^ Da (Supplementary Figure 1). Importantly, immunodetection of RpdA showed a retention profile similar to FscA, providing an independent confirmation of the TAP results (Supplementary Figure 1).

### ScrC Recruits FscA to the RcS2LF Complex

In order to investigate RcLS2F composition more precisely, *scrC* and *fscA* mutants in an RpdA^TAP^ strain were generated and used for purifications as described above. In the Δ*fscA* strain, all interaction partners apart from FscA were precipitated by the RpdA^TAP^ bait (Table 2). In the Δ*scrC* strain, however, neither ScrC nor FscA were identified in the RpdA TAP elution fraction (Table 2). This result indicates that ScrC directly recruits FscA to the RpdA complex and is critical for complete RcLS2F formation.

**TABLE 2:** ScrC recruits FscA to the RcLS2F complex.

		MW	Δ*fscA*	Δ*scrC*
*A. nidulans*	Accession	[kD]	RpdA TAP	RpdA TAP
ScrC	AN8823	92.7	57.5 (41)	
FscA	AN4022	83.3		
RpdA	AN4493	75.4	72.0 (53)	71.5 (51)
SinC	AN1546	179.5	70.5 (110)	69.8 (106)
PrwA	AN8187	46.2	69.0 (23)	57.5 (20)
SdsC	AN3178	55.6	65.0 (26)	56.0 (24)
LafA	AN5099	44.1	42.6 (15)	44.0 (15)

*Results of RpdA TAP in Δ*fscA* and Δ*scrC* backgrounds filtered for RcLS2F components are displayed. Refer to the caption of Table 1 for details and to Supplementary Data Sheet 1 for complete protein identification data.*

### RcLS2F Is a Nuclear Complex Exhibiting *in vitro* Histone Deacetylase Activity

**FIGURE 2:**
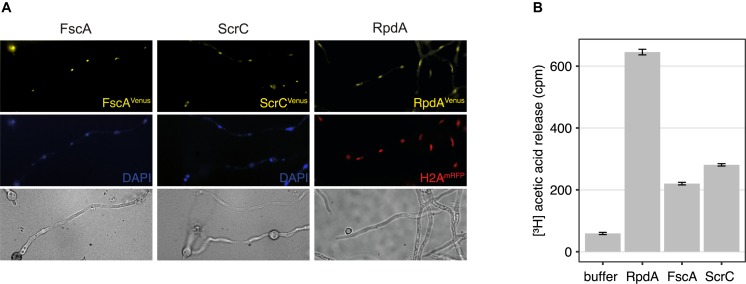
RcLS2F is a nuclear complex displaying *in vitro* KDAC activity. **(A)** Fluorescence microscopy of Venus-tagged versions under the control of the xylanase promoter induced by the addition of 1% xylose. Nuclei were stained by DAPI (FscA and ScrC) or by the introduction of a mRFP-tagged H2A allele (RpdA; Bayram et al., 2012a). **(B)** Histone deacetylase assay of IgG pull-downs.

To test the cellular localization of ScrC and FscA, strains expressing C-terminally Venus-tagged versions, ScrC^Ven^ and FscA^Ven^, under control of the tunable *Penicillium chrysogenum* xylanase promoter (Zadra et al., 2000; Tribus et al., 2010) were used for epifluorescence microscopy. These experiments revealed that, as shown for RpdA (Bauer et al., 2016), both proteins are mainly located within the nucleus (Figure 2A) confirming RcLS2F as a nuclear complex. In order to determine RcLS2F as catalytically active complex, we also used ScrC^TAP^ and FscA^TAP^ strains for IgG pull-downs. Both precipitates clearly showed deacetylase activity which amounts to about 30% of total RpdA activity that was pulled down by RpdA^TAP^ (Figure 2B). This result confirms histone deacetylase activity of RcLS2F *in vitro*.

### ScrC and FscA Are Conserved Fungal Proteins

**FIGURE 3:**
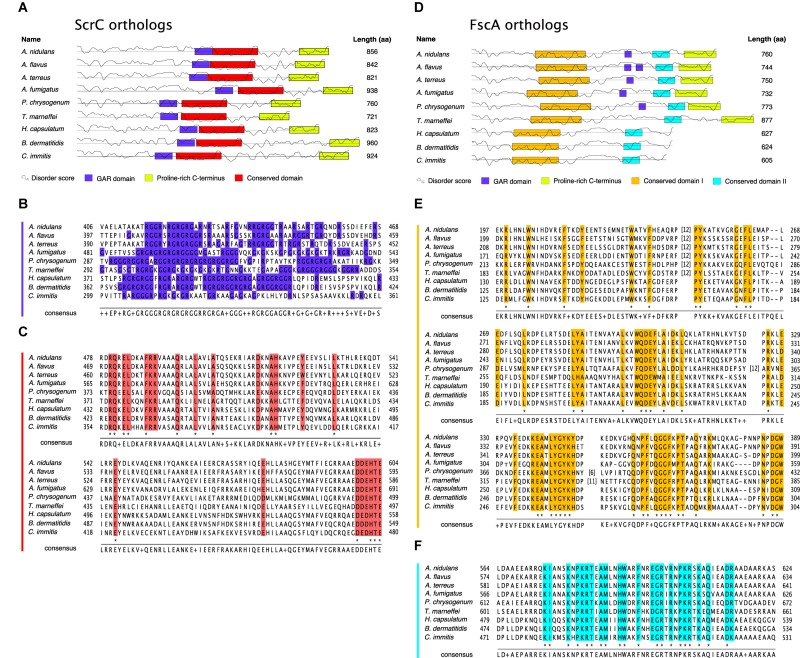
ScrC and FscA are conserved fungal proteins. Schematics of nine ScrC **(A)** and FscA **(D)** orthologs. Boxes display sequence motifs as identified by the MEME algorithm (conserved domains) or annotated manually. Straight lines indicate a residue disorder probability of 0.6, the curved lines represent the corresponding residue disorder score as calculated by PrDos. **(B)** Amino acid sequences in single letter code corresponding to the GAR domain of ScrC orthologs as labeled in **(A)**. Arg and Gly residues are labeled in blue. **(C)** Sequence alignment of the conserved domain of ScrC orthologs from **(A)**. Sequences shown below *A. nidulans* ScrC in **(B**,**C)** were extracted from: RAQ50577.1 (*A. flavus*); XP_001218051.1 (*A. terreus*); XP_754030.1 (*A. fumigatus*); KZN87476.1 (*P. chrysogenum*); KFX44415.1 (*T. marneffei*); EEH10392.1 (*H. capsulatum*); KMW69461.1 (*B. dermatitidis*); KMU80291.1 (*C. immitits*). **(E)** Sequence alignment of the conserved domain I of FscA orthologs from **(D)**. **(F)** Sequence alignment of the conserved domain II of FscA orthologs from **(D)**. Sequences shown below *A. nidulans* FscA in **(E**,**F)** were extracted from: RAQ42536.1 (*A. flavus*); XP_001269784.1 (*A. terreus*); EDP55714.1 (*A. fumigatus*); KZN83984.1 (*P. chrysogenum*); KFX42597.1 (*T. marneffei*); EGC46041.1 (*H. capsulatum*); EGE79043.1 (*B. dermatitidis*); XP_001245164.1 (*C. immitits*). Numbers (n) in square brackets indicate insertion of n residues at the respective sequence position. Colored columns indicate high (functional) conservation, residues conserved in all aligned sequences are marked by asterisks below sequence stacks. Consensus sequences shown below sequence stacks display the residues represented most often in the nine sequences at the respective position. Two or more residues occurring at the same frequency are displayed by “+.”

To determine the conservation of ScrC and FscA, BLASTp^1^ searches against available proteomes were performed. These analyses revealed that both proteins are conserved within the Ascomycetes subclade Eurotiomycetidae only, a fungal group containing a number of medically, biotechnologically, and agriculturally relevant genera such as *Aspergillus*, *Penicillium*, *Coccidioides*, or *Histoplasma*. As determined applying the fungiDB^2^ genome browser, both genes are arranged in similar syntenic surroundings. As searches of the Pfam database^3^ (El-Gebali et al., 2019) did not yield any known protein domains, orthologs of nine fungal species were used for MEME^4^ analysis (Bailey and Elkan, 1994). Interestingly, a putative conserved domain of 150 aa (Figures 3A,C) preceded by a glycine-arginine-rich (GAR) region (Figures 3A,B) was identified in ScrC orthologs. Alignment of FscA orthologs revealed presence of two conserved domains in all nine species (Figures 3D–F), a GAR region, however, could only be detected for *Aspergillus* and *Penicillium* orthologs (Figure 3D). Regions comprising a remarkably high proline content in both proteins (>35% within their 100 C-terminal residues) prompted us to use PrDOS^5^ (Ishida and Kinoshita, 2007) for prediction of intrinsically disordered regions. These analyses revealed that major fractions of the tested proteins have high disorder propensity (Figures 3A,D). Remarkably, major fractions of the residues below a disorder probability of 0.6 were identified within the indicated conserved domains (Figures 3A,D).

### The RcLS2F Complex Is Important for Normal Growth and Conidiation and Is Essential for Sexual Development

**FIGURE 4:**
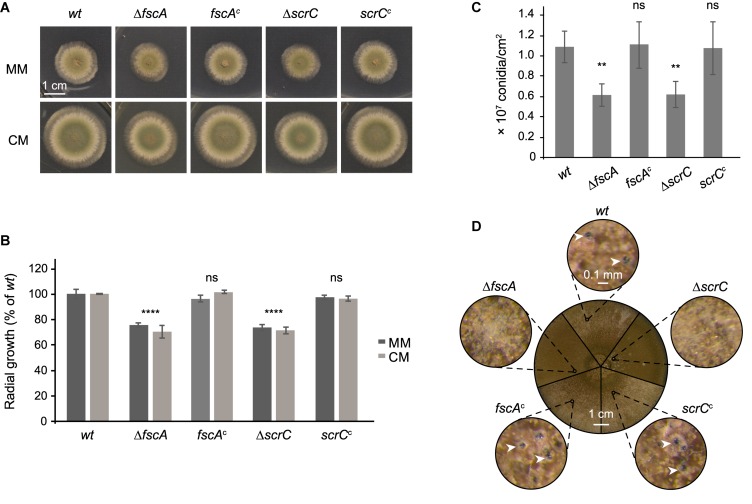
Δ*scrC* and Δ*fscA* strains display reduced radial growth and conidiation and are lacking sexual reproduction. Wild type, deletion, and complemented strains were dotted (1 × 10^4^ conidia each) onto MM and CM plates supplemented with arginine and biotin and incubated at 37°C. Unless otherwise indicated, MM was used as growth medium. **(A)** Morphology of representative colonies incubated for 48 h is shown. **(B)** Colony areas of strains grown on MM or CM for 48 h were quantified using Fiji (Schindelin et al., 2012). **(C)** Conidiation rates of wild type and mutant strains after growth for 7 days. Mean values in percent of wild type ± standard deviation of three biological replicates are displayed and statistical difference to the wild type was calculated by one-way ANOVA. *P < 0.05, **P < 0.01, ***P < 0.001, ****P < 0.0001, ns, not significant. **(D)** Sexual reproduction of wild type and mutant strains. Plates were sealed and incubated for 3 weeks in the dark. The circle in the center shows sectors of photographs of the plates of the different strains as indicated. Outer circles show details of the small circles in the center at 60×magnification. White arrow heads point to cleistothecia.

To further characterize FscA and ScrC, we generated deletion mutants by replacing the corresponding coding sequences with the *pyrG* auxotrophic marker. Mutant strains displayed normal colony morphology with reduced diameter compared to the wild type (Figure 4A). Radial growth was reduced to approximately 80% of the wild type in both mutants, regardless of the medium used (Figure 4B). Complementation by reintegration of genes coding for the expression of Venus-tagged ScrC and FscA at the respective deletion loci reverted the growth phenotypes (Figures 4A,B) and confirmed the functionality of the Venus-tagged versions. Moreover, also conidiation of the mutant strains was compared to that of the wild type. Δ*scrC* and Δ*fscA* strains showed a significant reduction of conidiation that was restored in the complemented strains (Figure 4C). Furthermore, selfing experiments were conducted to analyze, if lack of RcLS2F would interfere with the capability of sexual reproduction. Interestingly, both mutants were not able to form cleistothecia but instead, only produced empty nests of Hülle cells (Figure 4D). This phenotype was fully restored in the reconstituted strains (Figure 4D).

### Perturbation of the RcLS2F Complex Alleviates Δ*crzA* Phenotypes Under Stress Conditions

**FIGURE 5:**
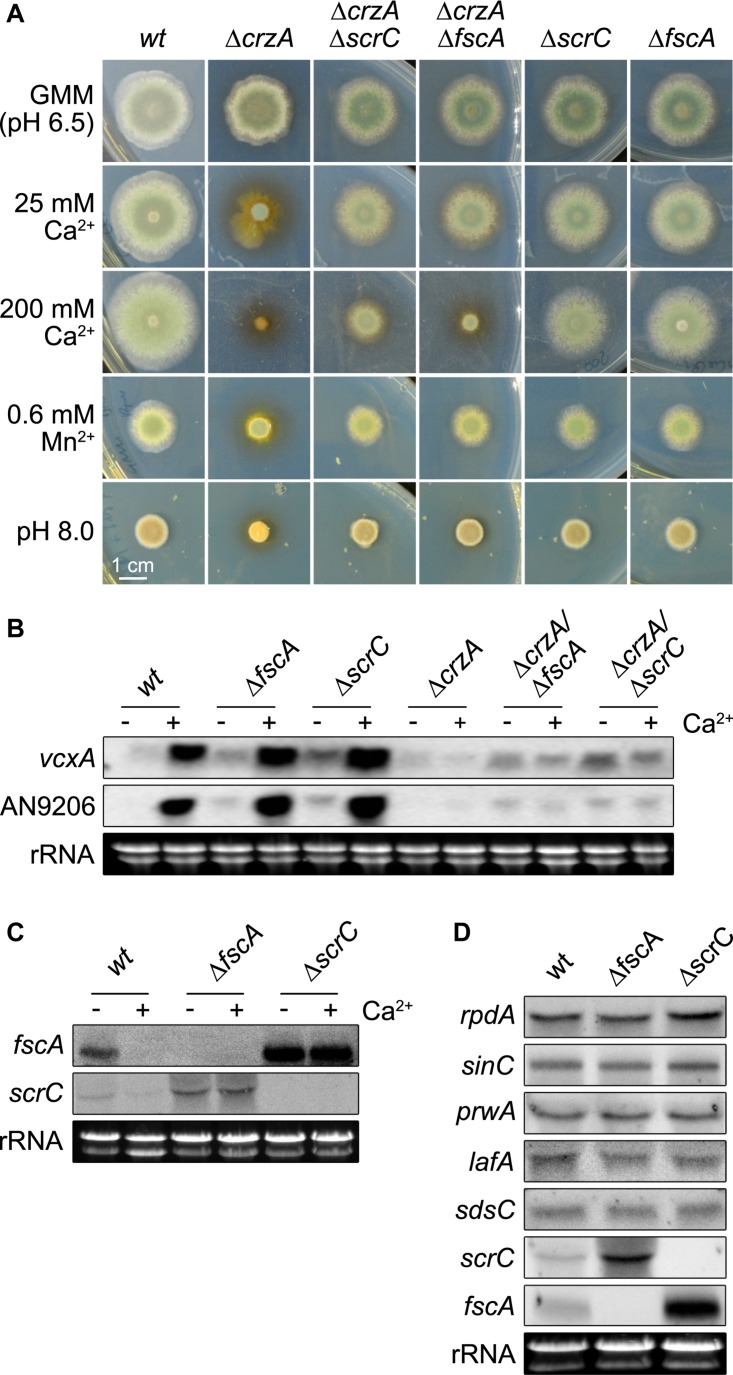
Deletion of *scrC* and *fscA* leads to Δ*crzA* bypass, de-repression of Ca^2+^-induced genes, and loss of autoregulation. **(A)** Conidia (2 × 10^3^) were point-inoculated and grown for 2 days at 37°C on MM including supplements as indicated. Representatives of at least three biological replicates are shown. Length of scale bar (1 cm) is displayed. Expression of *vcxA* and AN9206 **(B)** as well as of *fscA* and *scrC* **(C)** before and after exposure to Ca^2+^ (50 mM) for 20 min, and of RcLS2F complex members **(D)** was analyzed by northern analysis in wild type and mutant strains. Fungal strains (2 × 10^6^ conidia per ml) were grown in MM for 18 h prior induction. Expression of genes was determined using 10 μg of total RNA detected by DIG-labeled probes. Ethidium bromide stained rRNA was used as quality and loading control.

CrzA is the transcription factor targeted by the calcium (Ca^2+^) signaling cascade in fungi and its deletion becomes deleterious under several types of stresses, like exposure to Ca^2+^, manganese, or alkaline pH (Hagiwara et al., 2008; Spielvogel et al., 2008). In a Δ*crzA* suppressor screen, the RcLS2F-defining component ScrC was identified among others (Almeida et al., 2013). Comparable phenotypes of Δ*scrC* and Δ*fscA* as well as their mutual co-purification indicated similar functions within the RcLS2F complex. To examine this assumption in more detail, the coding sequence of *crzA* was deleted in a wild type, a Δ*scrC*, and a Δ*fscA* background and plate growth analyses were performed under Ca^2+^, manganese and alkaline pH stress. Intriguingly, both Δ*scrC* and Δ*fscA* strains attenuated the deleterious effects of the *crzA* deletion (Figure 5A). Interestingly and in contrast to low Ca^2+^, manganese, or pH, high Ca^2+^ revealed a reduced suppressive capacity of Δ*fscA* when compared to Δ*scrC* (Figure 5A), which is in line with the presumption of a recruitment of FscA via ScrC. Interestingly, calcium clearly increased radial growth in the wild type but neither in Δ*fscA* nor in Δ*scrC* strains (Figure 5A).

### RcLS2F Acts as Repressor of Ca^2+^-Induced Genes and Is Engaged in Autoregulation

**FIGURE 6:**
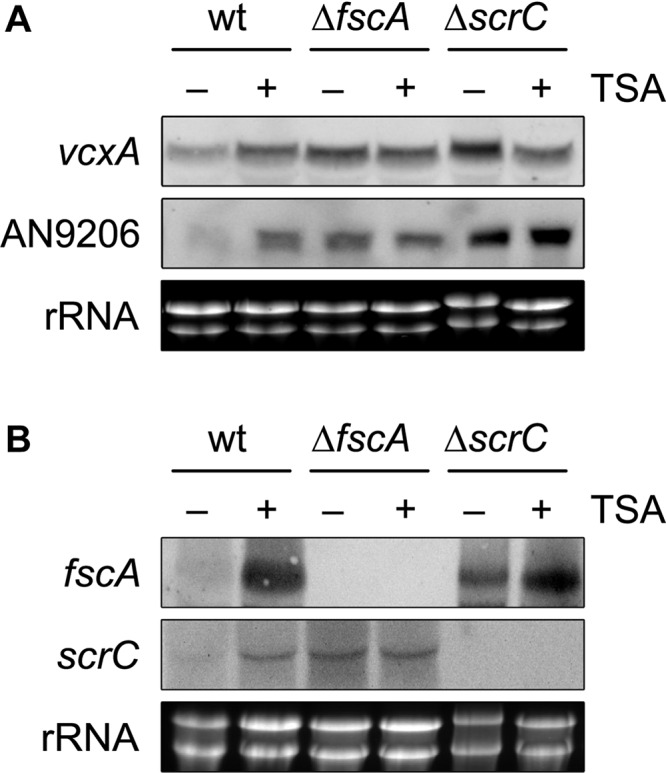
KDAC inhibition by TSA leads to de-repression of *crzA* targets and RcLS2F-specific genes. Expression of the CrzA targets *vcxA* and AN9206 **(A)** and of the RcLS2F components *scrC* and *fscA* **(B)** detected by northern analysis is displayed. Fungal strains (2 × 10^6^ conidia per ml) were grown in MM including appropriate supplements for 18 h and subsequently were challenged by the addition of 1 μM of Trichostatin A (TSA) for 30 min. Corresponding transcripts were visualized by northern analysis using 12.5 μg of total RNA and DIG-labeled probes. Ethidium bromide stained rRNA was used as quality and loading control.

The results shown above suggested a connection of the suppressor function of Δ*scrC* and Δ*fscA* via the RcLS2F complex. It was tempting to speculate that disruption of this nuclear complex might interfere with proper regulation of transcription. To test if the expression of known Ca^2+^-induced CrzA-dependent genes would be affected by disruption of RcLS2F, two genes were selected for northern analysis under Ca^2+^-induction: *vcxA*, a previously characterized vacuolar Ca^2+^/H^+^ exchanger (Spielvogel et al., 2008), and AN9206, a gene reported to be strongly induced by Ca^2+^ (Hagiwara et al., 2008). Ca^2+^ induction of *vcxA* and AN9206 was not affected by *scrC* or *fscA* deletion in the *crzA*+ background. In the absence of Ca^2+^, however, de-repression of both genes was observed in both mutants (Figures 5B, 6A). Densitometric quantification of northern blotting results revealed an increase of transcript abundance of 2.4/3.3 × and 3.0/4.4 × for *vcxA*/An9206 in Δ*fscA* and Δ*scrC* mutants, respectively (Supplementary Table 2). In the Δ*crzA* background, none of the strains could induce expression of these genes, but again both *vcxA* and AN9206 were clearly de-repressed in the RcLS2F deletion mutants (Figure 5B). Remarkably, release of repression generally was more pronounced in Δ*scrC*, which fits to the crucial role of ScrC in the assembly of the RcLS2F complex.

The observed antagonism between CrzA and ScrC/FscA raised the question if expression *scrC*/*fscA* themselves would be affected by Ca^2+^. Indeed, northern analysis showed that both *fscA* and *scrC* were transcriptionally repressed upon exposure to Ca^2+^ in the wild type. In the deletion strains, however, this repression was replaced by a strong up-regulation of *scrC* in Δ*fscA* and *vice versa*, indicating an autoregulatory feed-back loop epistatic to Ca^2+^-based repression (Figure 5C). We then examined if this autoregulation would affect other RcLS2F components as well. In contrast to *fscA* and *scrC*, however, expression of the remaining complex partners (*rpdA*, *sinC*, *prwA*, *sdsC*, *lafA*) was not affected in the two mutants (Figure 5D). Taken together, these results indicate transcriptional autoregulation of *scrC* and *fscA* and a RcLS2F-mediated repression of *vcxA* and AN9206 in the absence of Ca^2+^ stress conditions.

### Repression Defects Observed in *scrC* and *fscA* Mutants Are Functionally Linked to RcLS2F Deacetylase Activity

The lack of transcriptional repression observed in both RcLS2F mutants prompted us to investigate whether this regulation depends on RcLS2F deacetylase activity. Due to the pleiotropic phenotype of *rpdA* knock-down mutants resulting in severe growth defects, pharmacological inhibition of RpdA by trichostatin A (TSA) treatment was used to address this question. Indeed, TSA treatment for 30 min caused de-repression of *vcxA* and AN9206 in wild type mycelia comparable to that of the Δ*fscA* and slightly lower than that in the Δ*scrC* mutant (Figure 6A). Remarkably, TSA treatment also led to de-repression of *fscA* and *scrC* in the wild type (Figure 6B), which is in line with the observed autoregulation of RcLS2F (Figure 5B).

Taken together, our data suggest that the novel mold-specific RcLS2F KDAC complex, containing two poorly characterized proteins as defining and crucial components, is functionally implicated in Ca^2+^-mediated transcriptional regulation via its co-repressor activity.

## Discussion

Class 1 KDACs are known to act as multimeric complexes and therefore, targeting specific protein–protein interactions might be an alternative to the development of catalytic KDAC inhibitors (Millard et al., 2017). As the fungal class 1 KDAC RpdA was confirmed recently as promising anti-fungal target (Bauer et al., 2016, 2019a) knowledge of the composition of RpdA complexes is of utmost importance.

Using a TAP strategy and based on analogy to previously described RpdA-type complexes in baker’s and fission yeast, we provide evidence for three RpdA complexes in *A. nidulans*. All three complexes described contain a conserved core consisting of RpdA, SinC, and PrwA. So far, fungal class 1 KDAC/Sin3 complexes have been analyzed in yeasts, resulting in the characterization of two complexes, Rpd3L and Rpd3S in *S. cerevisiae* (Lechner et al., 2000; Carrozza et al., 2005b) and three versions of a large complex, Clr6I/Clr6I′/Clr6I′′, and one small complex, Clr6II, in *S. pombe* (Nicolas et al., 2007; Zilio et al., 2014). Recently, an RpdA-L-like complex has also been described in the fungal plant pathogen *Fusarium pseudograminearum* (Zhang et al., 2019).

While the function of these complexes has been analyzed in great detail in yeasts (see introduction), no functional role has been described for RpdA complexes yet. Nevertheless, RpdA depletion has been shown to activate defined silent gene clusters in *A. nidulans* (Albright et al., 2015).

The novel RcLS2F complex introduced in this paper contains five proteins, which are as well components of the RpdA-L complex. Clear orthologs of the complex-defining members ScrC and FscA, however, are present in the ascomycete class Eurotiomycetes only. Given that ScrC recruits FscA to the complex, it seems likely that lack of FscA results in a remnant RcLS2F complex whereas in case of ScrC loss, its formation is completely abolished. This assumption is in line with the fact that *fscA* deletion resulted in reduced suppressive capacity and milder de-regulation of the genes analyzed compared to *scrC* deletion.

The proteins Clr6 as well as Pst1 and Sds3, corresponding to *A. nidulans* RpdA, SinC, and SdsC, respectively, were shown to be crucial for viability in fission yeast (Grewal et al., 1998; Nicolas et al., 2007). Although, RpdA itself is essential for growth and development of *A. nidulans* and *A. fumigatus* (Tribus et al., 2010; Bauer et al., 2016), the deletion of *scrC* and *fscA* resulted in viable strains but with reduced conidiation and abolished sexual development. Viability of strains lacking RcLS2F, however, not necessarily implies that RcLS2F is not at all involved in the vital role of RpdA. Recent data from fission yeast indicate that the lethality of deletions of genes coding for Clr6I components could be rescued by deletion of those coding for Clr6II components (Li J. et al., 2019). It therefore was assumed that the detrimental effects caused by deletion of Clr6I might be the result of an unbalanced Clr6I/II complex equilibrium (Li J. et al., 2019). In order to exclude synthetic lethality of Δ*fscA* and Δ*scrC*, we also have generated double mutant strains that were viable and showed phenotypes comparable to Δ*fscA* and Δ*scrC* mutants (Supplementary Figure 2). These data are in line with the protein interaction results, which suggested recruitment of FscA by ScrC in the RcLS2F complex.

The central RcLS2F defining component ScrC was previously identified in a screen for suppressors of Δ*crzA* (Almeida et al., 2013). CrzA is the down-stream effector of the Ca^2+^ signaling cascade, which is conserved throughout eukaryotes (Cai et al., 2014). Mammalian counterparts to CrzA are the members of the NFAT transcription factor family (Rao et al., 1997). Ca^2+^ is an essential messenger via its interaction with calmodulin and the phosphatase calcineurin (Clapham, 2007). On the other hand, Ca^2+^ is one of the most restricted ions in the cytosol because of its toxicity at high concentrations (Espeso, 2016). In addition to elevated Ca^2+^ in the surrounding environment, specific stimuli including alkaline pH or heavy metal stress also lead to increased Ca^2+^ influx into the cytosol of fungi (Liu et al., 2015). Binding of Ca^2+^ to calmodulin then activates the calmodulin-calcineurin cascade that finally results in dephosphorylation and translocation of CrzA from the cytosol to the nucleus (Hernández-Ortiz and Espeso, 2013, 2017).

We suggest that lack of the RcLS2F complex is responsible for the described genetic bypass of Δ*crzA*. Two observations argue for this conclusion: on the one hand, we show that Δ*fscA* phenocopies the suppressor effect of Δ*scrC*, though to a lesser extent, which is in line with the recruitment of FscA by ScrC. The differences in suppressive capabilities might also explain the fact that only *scrC* but not *fscA* was identified in the above-mentioned suppressor screen. On the other hand, by pharmacological inhibition of the catalytic component RpdA with TSA, we recapitulated gene de-regulation as seen in RcLS2F mutants in the wild type, which indicates that catalytic activity of RcLS2F, i.e., RpdA, is critical for its repressor function. However, it seems likely that alleviation of Δ*crzA* under stress comes at the expense of a general reduction of fitness as indicated by radial growth reduction in the absence of stress conditions.

Binding of the transcription factor CrzA to specific DNA motifs results in the expression of Ca^2+^-response genes, for instance of the vacuolar Ca^2+^/H^+^ exchanger VcxA (Hagiwara et al., 2008; Spielvogel et al., 2008). One possibility for the alleviation of Ca^2+^ toxicity in strains lacking both CrzA and RcLS2F is an induction of Ca^2+^-dependent genes in the absence of CrzA. However, this was not the case, illustrating that the positive regulator CrzA is needed for full expression. Nevertheless, the observed increase in transcript levels of *vcxA* and AN9206 in the RcLS2F mutant strains without induction by Ca^2+^ suggests that their de-regulation plays a role in Δ*crzA* suppression. Notably, the extent of de-repression was mimicking the mode of recruitment to the complex, i.e., less de-repression in Δ*fscA* compared to Δ*scrC* strains.

**FIGURE 7:**
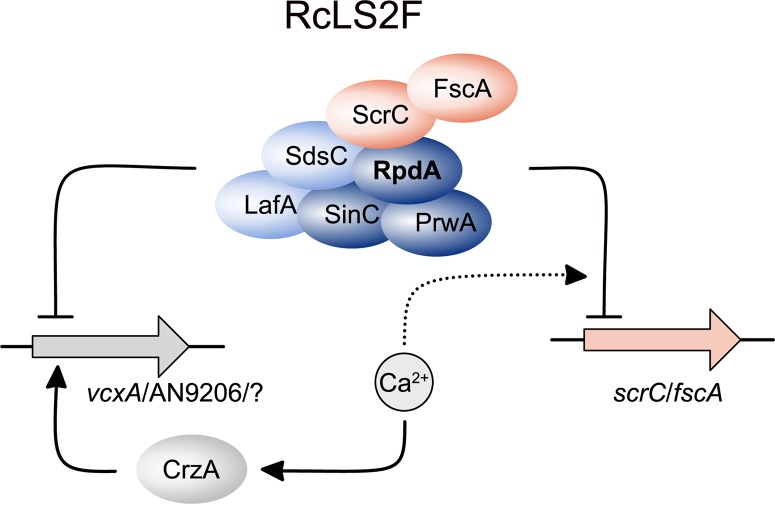
Schematic depiction summarizing the role of RcLS2F in autoregulation and repression of CrzA-dependent genes. Proteins are displayed as ellipses, genes as filled arrows. Lines with arrow heads and barred ends indicate activation and inhibition, respectively. Dashed lines with arrow heads and barred ends display positive and negative influence on regulatory events, respectively.

As summarized in Figure 7, lack of RcLS2F results in a de-regulation of genes up-regulated upon Ca^2+^ increase but without induction by Ca^2+^, as shown exemplarily for *vcxA* and AN9206. Although the loss of repression of these two genes alone may not be sufficient to explain the suppressor phenotypes of Δ*fscA* and Δ*scrC*, it is likely that the de-repression observed might hold true also for other genes involved in Ca^2+^ homeostasis. In this case, additive effects of several proteins expressed continuously at low levels could be sufficient to partially bypass loss of CrzA. The exact mechanism, however, remains to be studied. In line with the suggested RcLS2F function antagonizing CrzA-induced transcription of Ca^2+^ responsive genes, increasing Ca^2+^ levels result in repression of *fscA* and *scrC*. Moreover, the RcLS2F complex is involved in a deacetylase-dependent autoregulatory feed-back loop. This is in accordance with results described previously for the mammalian class 1 deacetylase HDAC1, where a similar feed-back loop was suggested (Schuettengruber et al., 2003).

## Materials and Methods

### Fungal Strains and Growth Media

Strains used in this study are listed in Supplementary Table 3. Strains were grown on glucose minimal medium [MM: 1% (m/v) glucose, 10 mM di-ammonium tartrate, salt solution, and trace elements] or glucose complete medium [CM: 2% (m/v) glucose, 0.2% (m/v) peptone, 0.1% (m/v) yeast extract, 0.1% (m/v) casamino acids, salt solution, and trace elements] as described (Cove, 1966). If not stated otherwise, MM was supplemented with 0.1 μg/ml biotin and 4 mM arginine. All other auxotrophs were supplemented as described elsewhere (Todd et al., 2007). Alleles driven by the *xylP* promoter were induced by addition of 0.05–1% of xylose to MM.

### Generation of Transformation Cassettes and Mutant Strains

For cloning, amplification, digestion, and propagation of DNA fragments and vectors, standard molecular techniques were used (Sambrook and Russell, 2001). Oligonucleotides and plasmids used for strain construction are listed in Supplementary Table 4. Cassettes for genetic manipulation were generated by PCR fusions as described (Shevchuk et al., 2004) or by In-Fusion HD Cloning (Clontech) in accordance with the manufacturer’s instructions. Expression constructs for FscA^Venus^ and ScrC^Venus^ were generated by cloning the respective coding sequences amplified from genomic DNA into *Nco*I and *Not*I sites of pIB97, which descends from pIB92 (Bauer et al., 2016) but with a functional *argB* allele. Fungal transformations were performed as described previously (Szewczyk et al., 2006) using an *nkuA* disruptant that was generated as follows. First, strain RIB1.22 harboring *biA*1, r*iboB*2, *pyrG*89, and *argB*2 alleles was isolated after genetic crossing (Todd et al., 2007). Next, the sequence coding for the C-terminus of NkuA was deleted using the *Af*_*riboB* marker. The resulting strain TIB24.12 was prone to homologous recombination due to a lack of functional NkuA and was used as recipient in subsequent transformations. Deletions of *fscA* and *scrC* were performed employing the *Af*_*pyrG* marker, *crzA* deletions with the *Af*_*bioDA* marker (Magliano et al., 2011). *A. fumigatus* (*Af*) orthologs were used as auxotrophic marker genes (*argB*, *bioDA*, *pyrG*, *riboB*) for transformation of non-homologous end-joining deficient strains.

For the complementation of *fscA* and *scrC*, a CRISPR-Cas9 system as described by Nødvig et al. (2015, 2018) was used to render the *Af*_*pyrG* marker non-functional by insertion of a premature termination codon (Supplementary Figure 3). The resulting *pyrG* auxotrophs were then transformed with cassettes composed of sequences encoding for C-terminally Venus-tagged versions of both deleted proteins, flanked by their 5′ UTR and part of the wild type *Af*_*pyrG* marker allele. By using a truncated *pyrG*, ectopic integration was excluded. *fscA*^Venus^*/scrC*^Venus^ fusions to *pyrG* were performed by PCR. To generate RpdA^TAP^ strains with *scrC* and *fscA* deletions, the *pyrG* auxtotroph ANOB486 was transformed with respective *Af_pyrG* marker constructs (see above). Transformants were confirmed by PCR screening and single integration of the deletion constructs was verified by Southern analysis as described elsewhere (Graessle et al., 2000).

### Tandem Affinity Purification (TAP)

Tandem affinity purification was performed as described (Bayram et al., 2012b; Bauer et al., 2019b). Briefly, C-terminally TAP-tagged strains were grown for 14 h in MM (including 0.05% xylose for TIB32.1) at 37°C and 180 rpm. Fungal mycelia were lyophilized and extracted as described (Bauer et al., 2019b). Purifications were monitored by western blotting using antibodies against RpdA or calmodulin binding protein and silver staining (Blum et al., 1987). For protein identification, samples were precipitated with TCA and then subjected to SDS-PAGE, together with a pre-stained protein ladder. To minimize separation of large proteins in the stacking gel, percentage was reduced to 3.5%. Shortly after the 300 kD marker band had left the stacking gel, electrophoresis was stopped and gels were stained with Coomassie brilliant blue. Gel slices from the lowest stained bands up to the end of the separating gel were used for in-gel trypsin digestion of the protein mixture followed by liquid chromatography-tandem mass spectrometry (LC-MS/MS).

### Liquid Chromatography Tandem Mass Spectrometry

**Method 1** (used for all identifications except RpdA TAP in TIB32.1).

Proteins in gel slices excised from SDS-PAGE gels were reduced with dithiothreitol, alkylated with iodoacetamide, and digested with trypsin from porcine pancreas (Promega) as described previously (Faserl et al., 2019). Tryptic digests were analyzed using an UltiMate 3000 RSCLnano-HPLC system coupled to a Q Exactive HF mass spectrometer (both Thermo Scientific), equipped with a Nanospray Flex ionization source. The peptides were separated on a homemade fritless fused-silica micro-capillary column (100 μm i.d. × 280 μm o.d. × 20 cm length) packed with 2.4 μm reversed-phase C18 material (Reprosil). Solvents for HPLC were 0.1% formic acid (solvent A) and 0.1% formic acid in 85% acetonitrile (solvent B). The gradient profile was as follows: 0–4 min, 4% B; 4–57 min, 4–35% B; 57–62 min, 35–100% B, and 62–67 min, 100% B. The flow rate was 300 nl/min.

The Q Exactive HF mass spectrometer was operated in the data dependent mode selecting the top 20 most abundant isotope patterns with charge state > 1 from the survey scan with an isolation window of 1.6 mass-to-charge ratio (m/z). Survey full scan MS spectra were acquired from 300 to 1750 m/z at a resolution of 60,000 with a maximum injection time (IT) of 120 ms, and automatic gain control (AGC) target 1 × 10^6^. The selected isotope patterns were fragmented by higher-energy collisional dissociation with normalized collision energy of 28 at a resolution of 30,000 with a maximum IT of 120 ms, and AGC target 5 × 10^5^.

Data Analysis was performed using Proteome Discoverer 2.1 (Thermo Scientific) with search engine Sequest. The raw files were searched against “FungiDB-36_AnidulansFGSCA4_ AnnotatedProteins” database (Galagan et al., 2005; Basenko et al., 2018). Precursor and fragment mass tolerance was set to 10 ppm and 0.02 Da, respectively, and up to two missed cleavages were allowed. Carbamidomethylation of cysteine was set as static modification and oxidation of methionine was set as variable modification. Acetylation, methionine-loss, and methionine-loss plus acetylation were set as N-terminal dynamic modification of proteins. Peptide identifications were filtered at 1% false discovery rate.

**Method 2** (used for initial RpdA TAP).

Protein bands were excised from gels and digested with trypsin from porcine pancreas (Sigma-Aldrich) as described previously (Faserl et al., 2019). Tryptic digests were analyzed using an UltiMate 3000 nano-HPLC system coupled to an LTQ Orbitrap XL mass spectrometer (both Thermo Scientific) equipped with a nanospray ionization source. The peptides were separated on a homemade fritless fused-silica microcapillary column (75 μm i.d. × 280 μm o.d. × 10 cm length) packed with 3 μm reversed-phase C18 material (Reprosil). Solvent for HPLC were 0.1% formic acid (solvent A) and 0.1% formic acid in 85% acetonitrile (solvent B). The gradient profile was as follows: 0–2 min, 4% B; 2–55 min, 4–50% B; 55–60 min, 50–100% B, and 60–65 min, 100% B. The flow rate was 250 nl/min.

The LTQ Orbitrap XL mass spectrometer was operated in the data dependent mode selecting the top 10 most abundant isotope patters with charge state 2 + and 3 + from the survey scan with an isolation window of 2 mass-to-charge ratio (m/z). Survey full scan MS spectra were acquired from 300 to 2000 m/z at a resolution of 60,000 with a maximum IT of 20 ms, and AGC target 1 × 10^6^. The selected isotope patterns were fragmented by collision induced dissociation (CID) with normalized collision energy of 35, and a maximum injection time of 55 ms.

Data analysis was performed using Proteome Discoverer 1.4 (Thermo Scientific) with search engine Sequest. The raw files were searched against “aspergillus_nidulans_ fgsc_a4_1_proteins” database (BROAD Institute; Galagan et al., 2005). Precursor and fragment mass tolerance was set to 10 ppm and 0.8 Da, respectively, and up to two missed cleavages were allowed. Carbamidomethylation of cysteine and oxidation of methionine were set as variable modifications. Peptide identifications were filtered at 1% false discovery rate.

### Western Analysis

Polyacrylamide gels were blotted onto nitrocellulose membrane using the Trans-Blot Turbo system (Bio-Rad). Blots were then blocked in TBS including 5% skim milk powder or 4% Ficoll 400 and probed with antibodies directed against RpdA (1:1300 dilution, Trojer et al., 2003), HdaA (1:1000 dilution, Trojer et al., 2003), GFP (Merck 11814460001, 1:5000 dilution), or CBP (Roche 07-482, 1:1333 dilution). Antibodies were detected with alkaline phosphatase conjugate antibodies directed against rabbit or mouse IgG (Sigma A3687 and A3562, respectively, 1:30000 dilution) and visualized using the BCIP/NBT colorimetric substrate (Promega S3771). Alternatively, primary antibodies were detected with fluorescent secondary antibodies (IRDye800/IRDye680, LI-COR Biosciences) according to the manufacturer’s instructions and visualized using the Odyssey imaging system (LI-COR Biosciences).

### Northern Analysis

If not otherwise stated, strains were grown for 18 h in MM with an inoculation density of 2 × 10^6^/ml. Total RNA was prepared from lyophilized fungal mycelia using TRI reagent (Sigma) according to the manufacturer’s instructions. RNA was electrophoresed in 1.2% agarose gels as described (Graessle et al., 2000) and then transferred to nylon membranes (Amersham Hybond-N) by down-ward capillary transfer (Chomczynski and Mackey, 1994). Digoxigenin-dUTP-labeled DNA probes specific for the analyzed transcripts were amplified with primers shown in Supplementary Table 4. Hybridized DNA probes were detected with alkaline phosphatase-conjugated anti-digoxigenin Fab fragments (Roche) and developed with CSPD chemiluminescent substrate (Roche) according to the manufacturer’s instructions. Signals were visualized with the Fusion-SL 3500 WL imaging system (Vilber Lourmat).

### Histone Deacetylase Activity Assay

Enzymatic activity of protein fractions was measured using [^3^H] acetate-labeled chicken histones as substrate as described (Bauer et al., 2019b). Briefly, fractions of 50 μl were mixed with 10 μl of labeled histones (4 mg/ml) and incubated at 25°C for 60 min. The reactions were stopped and extracted with ethylacetate. The organic phase was counted in an AccuFLEX LSC-8000 (HITACHI) liquid scintillation counter.

### Subcellular Localization of Proteins

To determine the subcellular localization of FscA, ScrC, and RpdA, strains expressing Venus-tagged proteins (Gsaller et al., 2014) were grown on cover slips in 6 well plates at 30°C overnight under conditions of induction of the *xylP* promoter (1% xylose). A strain expressing RpdA-Venus under the control of the xylanase promoter (TIB92n1; Bauer et al., 2016) was used as reference. Chromatin was stained with DAPI or by RFP-tagged H2A (Bayram et al., 2012a).

### Microscopy and Imaging

To monitor fungal growth on solid medium, images of samples were taken with a Nikon D5100 SLR camera or a Leica MZ16 stereomicroscope (Leica Microsystems GmbH) equipped with an AxioCam MRc camera (Carl Zeiss GmbH). For fluorescence microscopy, an Axioplan microscope (Carl Zeiss GmbH), equipped with (excitation/emission filters 365/420 nm for blue fluorescence, 500/535 nm for yellow fluorescence, and 565/620 nm for red fluorescence, Carl Zeiss GmbH) was used for imaging.

Image processing and editing was achieved with the programs Axio Vision (Carl Zeiss GmbH), the ImageJ distribution Fiji (Schindelin et al., 2012; Schneider et al., 2012), Affinity Photo (Serif, Inc.), and Affinity Designer (Serif, Inc.).

## Data Availability Statement

All datasets generated for this study are included in the article/Supplementary Material.

## Author Contributions

IB, SiG, PM, LK, BK, ÖSB, UB, FG, and BA performed the experiments. IB, ÖB, GB, and StG conceived and designed the experiments and wrote the manuscript. IB, LK, HL, ÖB, GB, and StG analyzed the data.

## Conflict of Interest

The authors declare that the research was conducted in the absence of any commercial or financial relationships that could be construed as a potential conflict of interest.

## Footnotes

1 https://blast.ncbi.nlm.nih.gov/Blast.cgi

2 https://fungidb.org

3 https://pfam.xfam.org

4 http://meme-suite.org

5 http://prdos.hgc.jp
