# Supplementary figures and images for: RcLS2F – A Novel Fungal Class 1 KDAC Co-repressor Complex in Aspergillus nidulans
Source: Front Microbiol. 2020 Feb 4;11:43. doi: 10.3389/fmicb.2020.00043 (PMC7010864; doi:10.3389/fmicb.2020.00043)

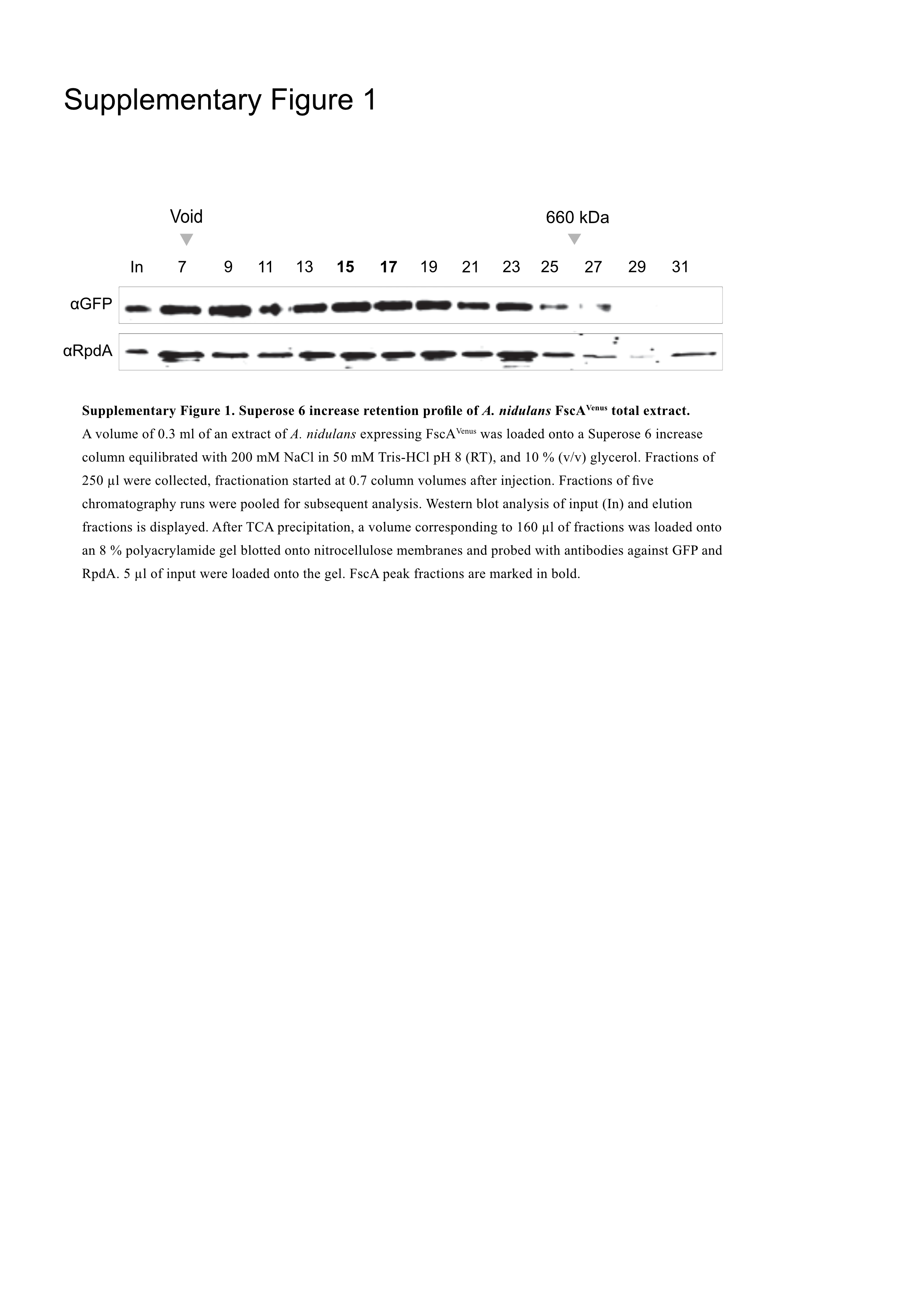

Supplement: Supplementary file 2 [file Image_1.JPEG]

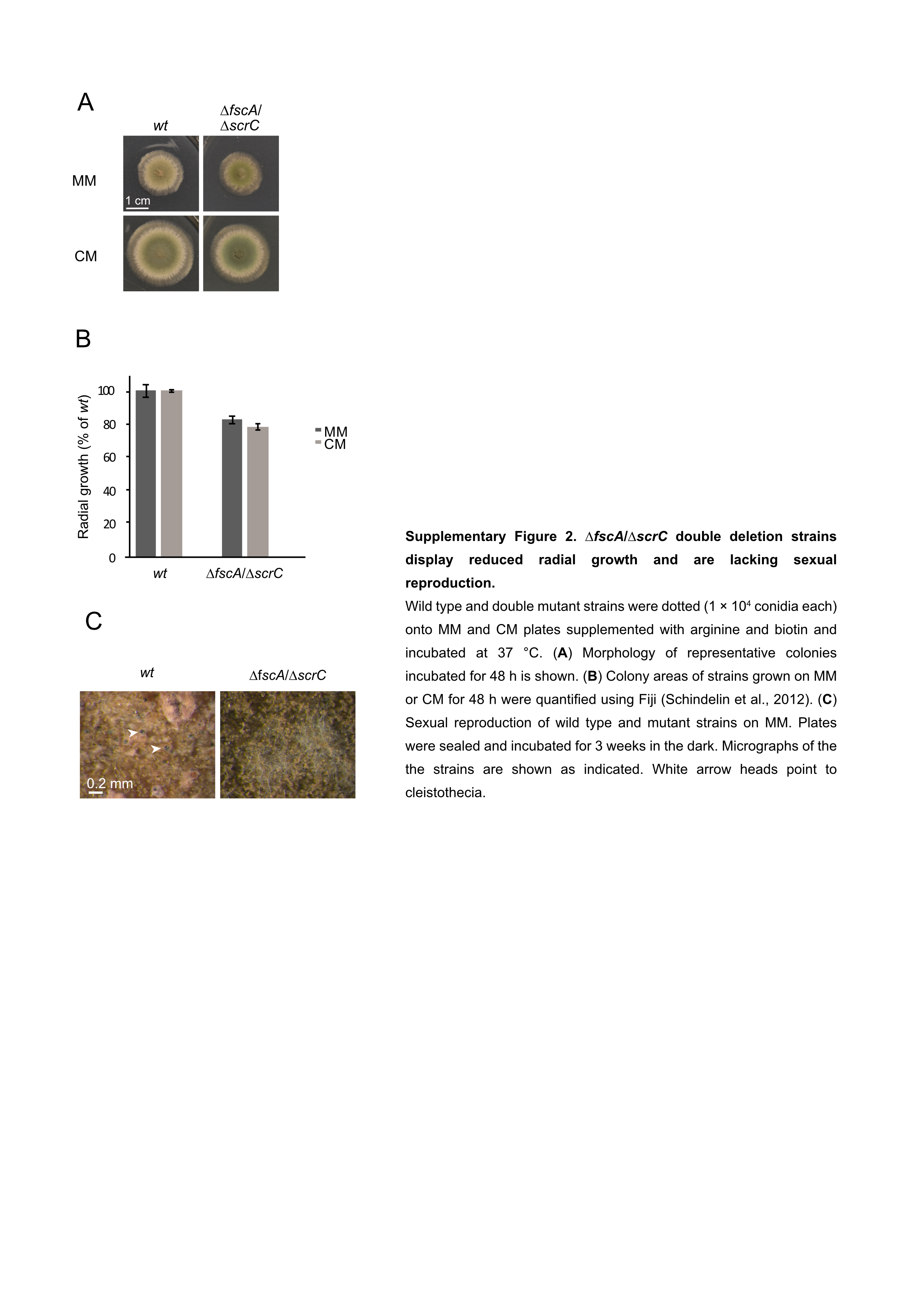

Supplement: Supplementary file 3 [file Image_2.JPEG]

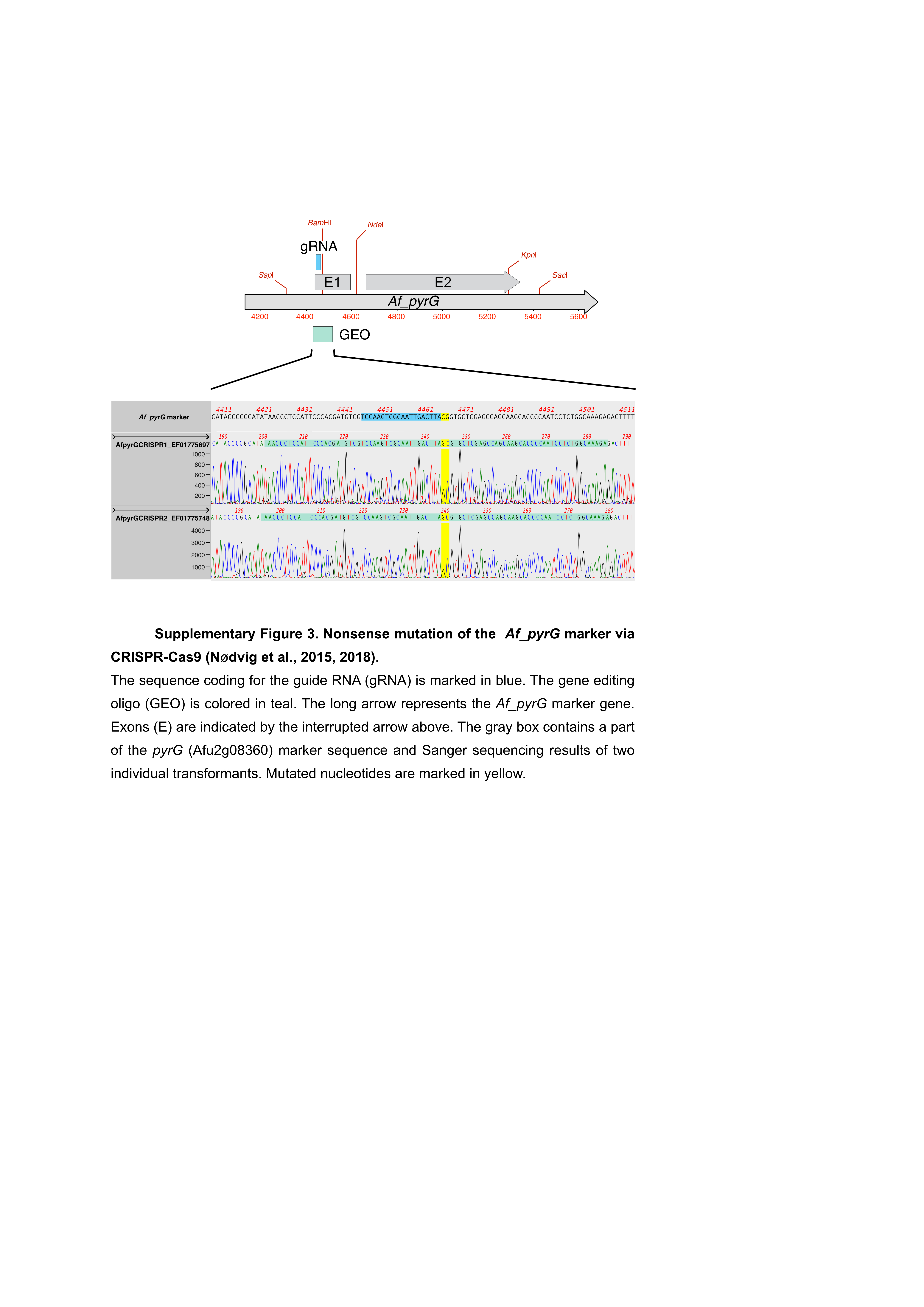

Supplement: Supplementary file 4 [file Image_3.jpg]
