# Supplementary material for: RcLS2F – A Novel Fungal Class 1 KDAC Co-repressor Complex in Aspergillus nidulans
Source: Front Microbiol. 2020 Feb 4;11:43. doi: 10.3389/fmicb.2020.00043 (PMC7010864; doi:10.3389/fmicb.2020.00043)
Supplement: Supplementary file 5 [file Table_1.DOCX]

**Supplementary Table 1. Putative *A. nidulans* RpdA complexes.**
Proteins in RpdA TAP fractions were identified by LC-MS/MS followed by BLASTp searches against *S. cerevisiae* and *S. pombe* databases. Subunits of yeast complexes are listed as defined previously (Lechner et al., 2000; Carrozza et al., 2005; Baker et al., 2013; Nicolas et al., 2007; Zilio et al., 2014). Molecular weight (MW) of *A. nidulans* proteins is indicated in kilodalton (kD). Mean values of sequence coverage (^a^) and identified unique peptides (^b^) of two independent purifications are shown (RpdA TAP). Empty fields indicate absence of respective proteins or detection below the limits. Refer to **Supplementary Data Sheet 1** for complete protein identification data. Absence of a clear ortholog in the *A. nidulans* proteome is indicated by “n/o”.

| Complex | Accession | *A. nidulans* | *S. cerevisiae* | *S. pombe* | MW [kD] | RpdA TAP |
| --- | --- | --- | --- | --- | --- | --- |
| RpdA-L | AN4493 | RpdA | Rpd3 | Clr6 | 75.4 | 49.3^a^ (31^b^) |
|  | AN1546 | SinC | Sin3 | Pst1 | 179.5 | 38.2 (46) |
|  | AN8187 | PrwA | Ume1 | Prw1 | 46.2 | 30.6 (9) |
|  | AN3178 | SdsC | Sds3 | Sds3 | 55.6 | 38.9 (13) |
|  | AN1375 | RxtB | Rxt2 | Rxt2 | 58.0 | 24.7 (11) |
|  | AN6280 | RxtC | Rxt3 | Rxt3 | 100.1 | 19.9 (15) |
|  | AN6196 | SapA | Sap30 |  | 24.4 | 59.1 (11) |
|  | AN5570 | PhoW | Pho23 | Png2 | 67.8 | 35.7 (15) |
|  | AN1453 | DepA | Dep1 | Dep1 | 75.1 | 21.9 (12) |
|  | AN4694 | CtiF | Cti6 | Cti6 | 67.4 | 10.2 (5) |
|  | n/o |  | Ume6 |  |  |  |
|  | n/o |  | Ash1 |  |  |  |
|  | AN5099 | LafA |  | Laf1/2 | 44.1 | 10.7 (3) |
|  | n/o |  |  | Nts1 |  |  |
|  | n/o |  |  | Mug165 |  |  |
|  | n/o |  |  | Png3 |  |  |
|  |  |  |  |  |  |  |
| RpdA-S | AN4493 | RpdA | Rpd3 | Clr6 | 75.4 | 49.3 (31) |
|  | AN1546 | SinC | Sin3 | Pst2 | 179.5 | 38.2 (46) |
|  | AN8187 | PrwA | Ume1 | Prw1 | 46.2 | 30.6 (9) |
|  | AN1976 | AlpM | Eaf3 | Alp13 | 38.1 | 40.4 (11) |
|  | AN7300 | CphB | Rco1 | Cph1/2 | 92.4 | 10.5 (6) |
|  |  |  |  |  |  |  |
| SntB | AN4493 | RpdA | Rpd3 | Clr6 | 75.4 | 49.3 (31) |
|  | AN8211 | KdmB^c^ | Ecm5 | Lid2 | 193.1 | 18.8 (20) |
|  | AN9517 | SntB | Snt2 | Snt2 | 56.6 | 23.9 (34) |

^a^ Mean values of sequence coverage of two independent purifications.

^b^ Mean values of number of identified unique peptides of two independent purifications.

^c^ KdmB is not the ortholog of Ecm5 but of Lid2 (Pfannenstiel et al., 2018).
