# Supplementary material for: RcLS2F – A Novel Fungal Class 1 KDAC Co-repressor Complex in Aspergillus nidulans
Source: Front Microbiol. 2020 Feb 4;11:43. doi: 10.3389/fmicb.2020.00043 (PMC7010864; doi:10.3389/fmicb.2020.00043)
Supplement: Supplementary file 6 [file Table_2.DOCX]

|  | *vcxA* | | AN9206 | |
| --- | --- | --- | --- | --- |
|  | mean | SD | mean | SD |
| ∆*fscA* | 2.39 | 0.65 | 3.33 | 1.35 |
| ∆*scrC* | 2.95 | 1.17 | 4.44 | 2.63 |

**Supplementary Table 2. De-repression of *vcxA* and AN9206 in ∆*fscA* and ∆*scrC* mutants.**Mean fold change values of mutants versus wild type are displayed. Corresponding standard deviations (SD) of five northern blots normalized against rRNA loading controls were calculated. Signals were quantified densitometrically using Fiji/ImageJ software.
