# Supplementary material for: RcLS2F – A Novel Fungal Class 1 KDAC Co-repressor Complex in Aspergillus nidulans
Source: Front Microbiol. 2020 Feb 4;11:43. doi: 10.3389/fmicb.2020.00043 (PMC7010864; doi:10.3389/fmicb.2020.00043)
Supplement: Supplementary file 7 [file Table_3.DOCX]

**Supplementary Table 3**: **Genotypes of *Aspergillus nidulans* strains used in this study**

| ***Strain*** | ***Ref. name study*** | ***Recipient*** | ***Variation with regard to recipient*** | ***Genotype*** | ***Reference*** |
| --- | --- | --- | --- | --- | --- |
| strains used for experiments | | | | | |
| TIB32.1 | RpdA^TAP^ | – | regulable *rpdA* TAP fusion | *alcA(p)*:*rpdA*; *xylP(p)*:*rpdA*:*TAP*; *alcA(p)*:*rpdA*; *veA1*; *argB2*; *yA2*; *pIB32*:*argB*; ArgB^+^; PyrG^+^ | Bauer *et al*. 2016 |
| TSG13.8 | FscA^TAP^ | TN02A3 | *fscA* TAP fusion | *fscA*:*TAP*:*Af_pyrG*; *∆nkuA*::*argB*; *pyroA4*; *veA1*; *pyrG89*; *ArgB+ PyrG+* | this study |
| TSG14.3 | ScrC^TAP^ | TN02A3 | *scrC* TAP fusion | *scrC*:TAP:*Af_pyrG*; ∆*nkuA*::*argB*; *pyroA4*; *veA1*; *pyrG89*; ArgB+ PyrG+ | this study |
| TIB15.1 | RpdA^TAP^/ ∆*fscA* | ANOB486 | *rpdA* TAP fusion; *fscA* deletion | *rpdA*:TAP:*natR*; ∆*fscA*::*Af_pyrG* ; *pyrG89* ; *pyroA4* ; ∆*nkuA*; PyrG+ | this study |
| TIB16.1 | RpdA^TAP^/ ∆*scrC* | ANOB486 | *rpdA* TAP fusion; *scrC* deletion | *rpdA*:TAP:*natR*; ∆*fscA*::*Af_pyrG* ; *pyrG89* ; *pyroA4* ; ∆*nkuA*; PyrG+ | this study |
| TIB26.1 | wild type | TIB24.12 | *pyrG89* allele complemented | *pyrG89*::*An*_*pyrG*; ∆*nkuA*(Cter)::*Af*_*riboB*; *pyrG89*; *riboB2*; *biA1*; *argB2*; RiboB^+^; PyrG^+^ | this study |
| TIB28.1 | ∆*fscA* | TIB24.12 | *fscA* deletion | ∆*fscA*::*Af*_*pyrG*; ∆*nkuA*(Cter)::*Af*_*riboB*; *pyrG89*; *riboB2*; *biA1*; *argB2*; RiboB^+^; PyrG^+^ | this study |
| TIB29.2 | ∆*scrC* | TIB24.12 | *scrC* deletion | ∆*scrC*::*Af*_*pyrG*; ∆*nkuA*(Cter)::*Af*_*riboB*; *pyrG89*; *riboB2*; *biA1*; *argB2*; RiboB^+^; PyrG^+^ | this study |
| TIB37.1 | ∆*fscA*/∆*scrC* | TIB28.1 | *fscA*/*scrC* deletion | ∆*fscA*::*Af*_*pyrG*; ∆*scrC*::*Af*_*argB*; ∆*nkuA*(Cter)::*Af*_*riboB*; *pyrG89*; *riboB2*; *biA1*; *argB2*; RiboB^+^; PyrG^+^; ArgB^+^ | this study |
| TIB45.3 | ∆*crzA* | TIB26.1 | *crzA* deletion | ∆*crzA*::*Af*_*bioDA*; *pyrG89*::An_*pyrG*; ∆*nkuA*(Cter)::*Af*_*riboB*; *pyrG89*; *riboB2*; *biA1*; *argB2*; RiboB^+^; BioDA^+^ | this study |
| TIB46.3 | ∆*fscA*/∆*crzA* | TIB28.1 | *fscA*/*crzA* deletion | ∆*fscA*::*Af*_*pyrG*; ∆*crzA*::*Af*_*bioDA*; ∆*nkuA*(Cter)::*Af*_*riboB*; *pyrG89*; *riboB2*; *biA1*; *argB2*; RiboB^+^; PyrG^+^; BioDA^+^ | this study |
| TIB47.3 | ∆*scrC*/∆*crzA* | TIB29.2 | *scrC*/∆*crzA* deletion | ∆*scrC*::*Af*_*pyrG*; ∆*crzA*::*Af*_*bioDA*; ∆*nkuA*(Cter)::*Af*_*riboB*; *pyrG89*; *riboB2*; *biA1*; *argB2*; RiboB^+^; PyrG^+^; BioDA^+^ | this study |
| TIB49.6 | *xylP*(p):*rpdA* | TIB24.12 | regulable *rpdA* | *rpdA*(p)::*Af*_*pyrG*; *xylP*(p):*rpdA*; ∆*nkuA*(Cter)::*Af*_*riboB*; *pyrG89*; *riboB2*; *biA1*; *argB2*; RiboB^+^; PyrG^+^ | this study |
| TIB50.2 | *xylP*(p):*rpdA*/ ∆*crzA* | TIB24.12 | regulable *rpdA*; *crzA* deletion | *rpdA*(p)::*Af*_*pyrG*; *xylP*(p):*rpdA*; ∆*crzA*::*Af*_*bioDA*; ∆*nkuA*(Cter)::*Af*_*riboB*; *pyrG89*; *riboB2*; *biA1*; *argB2*; RiboB^+^; PyrG^+^; BioDA^+^ | this study |
| TIB60.1 | *fscA*^c^ | TIB40 | ∆*fscA* complemented | ∆*fscA*::*Af*_*pyrG*_27::*fscA*:Venus:*Af*_*pyrG*; ∆*nkuA*(Cter)::*Af*_*riboB*; *pyrG89*; *riboB2*; *biA1*; *argB2*; RiboB^+^; PyrG^+^ | this study |
| TIB61.2 | *scrC*^c^ | TIB41 | ∆*scrC* complemented | ∆*scrC*::*Af*_*pyrG*_27::*scrC*:Venus:*Af*_*pyrG*; ∆*nkuA*(Cter)::*Af*_*riboB*; *pyrG89*; *riboB2*; *biA1*; *argB2*; RiboB^+^; PyrG^+^ | this study |
| TIB99.1 | FscA^Venus^ | A89 | regulable *fscA* Venus fusion | *biA1*; *argB2*; *veA1*; pIB99; ArgB^+^ | this study |
| TIB100.1 | ScrC^Venu^s | A89 | regulable *scrC* Venus fusion | *biA1*; *argB2*; *veA1*; pIB100; ArgB^+^ |  |
| TIB92n1 | RpdA^Venus^ | – | regulable *rpdA* Venus fusion | *rpdA*(p)::*Af*_*pyrG*; *alcA*(p):*rpdA*; *veA1*; *argB2*; *yA2*; pIB92:*argB*; pME3857; ArgB+; PyrG+; BleR+ | Bauer *et al*. 2016 |
|  |  |  |  |  |  |
| strains used for strain generation | | | | | |
| A89 |  |  |  | *biA1*; *argB2* | FGSC |
| TN02A3 |  |  |  | *pyrG89*; *argB2 ∆nkuA*::*argB*; *pyroA4 veA1* | Hoffmann *et al.* 2001 |
| ANOB486 |  |  | *rpdA* TAP fusion | *rpdA*:TAP:*natR*; *pyrG89;* *pyroA4*; ∆*nkuA* | Ö. Bayram |
| TIB7.6 |  |  |  | ∆*rmtA*::*argB*; *biA1*; *argB2*; *veA1*; ArgB+ | Bauer *et al*. 2019 |
| RHG11 |  | XA28 𝓧^a^ TIB7.6 |  | *yA2*; *riboB2*; *pyrG89* | Bauer *et al*. 2019 |
| RIB1.22 |  |  |  | *biA1*; *argB2*; *pyrG89*; *veA1*; *riboB2* | this study |
| TIB24.12 |  | RIB1.22 | *nkuA* truncation | ∆*nkuA*(Cter)::*Af*_*riboB*; *pyrG89*; *riboB2*; *biA1*; *argB2*; RiboB^+^ | this study |
| TIB40 |  | TIB28.1 | nonsense mutant *Af*_*pyrG* marker | ∆*fscA*::*Af*_*pyrG*_27^b^; ∆*nkuA*(Cter)::*Af*_*riboB*; *pyrG89*; *riboB2*; *biA1*; *argB2*; RiboB^+^; PyrG^-^ | this study |
| TIB41 |  | TIB29.2 | nonsense mutant *Af*_*pyrG* marker | ∆*scrC*::*Af*_*pyrG*_27; ∆*nkuA*(Cter)::*Af*_*riboB*; *pyrG89*; *riboB2*; *biA1*; *argB2*; RiboB^+^; PyrG^-^ | this study |

^a^indicates sexual crossing between strains

^b^premature termination codon inserted into *Af*_*pyrG* marker allele via CRISPR-Cas9
